# Precision targeting of the plasminogen activator inhibitor‐1 mechanism increases efficacy of fibrinolytic therapy in empyema

**DOI:** 10.14814/phy2.14861

**Published:** 2021-05-15

**Authors:** Galina Florova, René A. Girard, Ali O. Azghani, Krishna Sarva, Ann Buchanan, Sophia Karandashova, Christian J. DeVera, Danna Morris, Mignote Chamiso, Kathleen Koenig, Douglas B. Cines, Steven Idell, Andrey A. Komissarov

**Affiliations:** ^1^ The Department of Cellular and Molecular Biology of the University of Texas Health Science Center at Tyler (UTHSCT) Tyler TX USA; ^2^ The University of Texas at Tyler Tyler TX USA; ^3^ UTHSCT Vivarium Tyler TX USA; ^4^ Department of Pathology and Laboratory Medicine Perelman‐University of Pennsylvania School of Medicine Philadelphia PA USA

**Keywords:** empyema, fibrinolytic therapy, molecular target, plasminogen activator inhibitor‐1, single chain tissue plasminogen activator

## Abstract

Plasminogen activator inhibitor‐1 (PAI‐1) is an endogenous irreversible inhibitor of tissue‐type (tPA) and urokinase (uPA) plasminogen activators. PAI‐1‐targeted fibrinolytic therapy (PAI‐1‐TFT) is designed to decrease the therapeutic dose of tPA and uPA, attenuating the risk of bleeding and other complications. Docking site peptide (DSP) mimics the part of the PAI‐1 reactive center loop that interacts with plasminogen activators, thereby affecting the PAI‐1 mechanism. We used DSP for PAI‐1‐TFT in two rabbit models: chemically induced pleural injury and *Streptococcus pneumoniae* induced empyema. These models feature different levels of inflammation and PAI‐1 expression. PAI‐1‐TFT with DSP (2.0 mg/kg) converted ineffective doses of single chain (sc) tPA (72.5 µg/kg) and scuPA (62.5 µg/kg) into effective ones in chemically induced pleural injury. DSP (2.0 mg/kg) was ineffective in *S*. *pneumoniae* empyema, where the level of PAI‐1 is an order of magnitude higher. DSP dose escalation to 8.0 mg/kg resulted in effective PAI‐1‐TFT with 0.25 mg/kg sctPA (1/8th of the effective dose of sctPA alone) in empyema. There was no increase in the efficacy of scuPA. PAI‐1‐TFT with DSP increases the efficacy of fibrinolytic therapy up to 8‐fold in chemically induced (sctPA and scuPA) and infectious (sctPA) pleural injury in rabbits. PAI‐1 is a valid molecular target in our model of *S*. *pneumoniae* empyema in rabbits, which closely recapitulates key characteristics of empyema in humans. Low‐dose PAI‐1‐TFT is a novel interventional strategy that offers the potential to improve fibrinolytic therapy for empyema in clinical practice.

## Graphical abstract

Empyema remains an important clinical problem with high mortality among patients 65 years and older. Targeting plasminogen activator inhibitor 1 (PAI‐1) in two rabbit models of pleural injury resulted in to up to eight‐fold increases in the efficacy of fibrinolytic therapy (decrease in dose needed for effective treatment). Low‐dose PAI‐1‐targeting fibrinolytic therapy would expand the population of patients that could benefit from treatment and improve therapeutic outcomes.

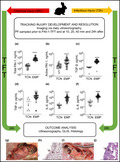

## 1 INTRODUCTION

Plasminogen Activator Inhibitor‐1 (PAI‐1) is a mechanism‐based inhibitor of tissue type (tPA) and urokinase (uPA) plasminogen activators (PAs) (Carrell et al., 1991; Chaillan‐Huntington et al., 1997; Mourik et al., 1984) used for fibrinolytic therapy. Levels of PAI‐1 increase by up to three orders of magnitude in empyema (pleural sepsis), and organizing pleural injury (Aleman et al., 2003; Chung et al., 2005; Philip‐Joet et al., 1995), limiting endogenous and exogenous plasminogen activating activity that could help prevent or treat uncontrolled fibrin deposition induced by inflammation. Previously, we suggested that PAI‐1 is a molecular target in treatment of chemically (tetracycline; TCN)‐induced pleural injury in rabbits (Karandashova et al., 2013). PAI‐1‐targeted fibrinolytic therapy (PAI‐1‐TFT) is designed to modulate the PAI‐1 mechanism in order to decrease the minimal effective dose (MED) that is a minimal dose of a plasminogen activator resulting in successful therapy for every animal in a group. We used a novel model of pleural injury induced by *Streptococcus pneumoniae* (Komissarov et al., 2016) to recapitulate the inflammation and high levels of PAI‐1 associated with the development of empyema in humans and its progression from the early, acute stage to more advanced chronic stages (Komissarov et al., 2016). Both single chain (sc) tPA and scuPA in a bolus MED 2.0 mg/kg are effective in clearing the pleural space in rabbits with acute *S*. *pneumoniae* empyema (Komissarov et al., 2016). The MED for empyema is 4‐ and 13.8‐fold higher than MEDs of scuPA and sctPA in TCN‐induced pleural injury in rabbits (Idell et al., 2007). This increase in the MED in *S*. *pneumoniae* empyema could reflect changes in the molecular mechanism of intrapleural fibrinolysis due to: (a) a slower rate of fibrinolysis, (b) faster *in vivo* inactivation of plasminogen activators, (c) an increase in the intrapleural level of PAI‐1, or a combination of these factors. We reasoned that if PAI‐1 is a true molecular target for pleural injury then PAI‐1‐TFT should reduce the MED of plasminogen activators in both chemical and infectious pleural injury, and chose to target the docking site (DS) of PAI‐1. uPA and tPA with alanine mutations of positively charged residues in the 37‐loop, which participates in exosite interactions (Coombs et al., 1998; Madison et al., 1990) (ΔDS‐uPA and tenecteplase, respectively), react with PAI‐1 at a rate that is almost two orders of magnitude slower than the WT enzymes (Benedict et al., 1995; Florova et al., 2018; Keyt et al., 1994). Fibrinolytic therapy with ΔDS‐scuPA in TCN‐induced pleural injury in rabbits showed a trend towards improved outcomes compared to WT‐ scuPA (Florova et al., 2018), indicating that targeting DS interactions could increase the efficacy of fibrinolytic therapy, although its success was limited by its short intrapleural half‐life. These results provide a rationale to further explore precision targeting of DS interactions with a PAI‐1 derived Docking Site Peptide (DSP; EEIIMD), which was used successfully in other translational research (Armstead et al., 2010a, 2010b). Targeting PAI‐1 mechanism with mAbs that redirect the reaction with plasminogen activator towards the substrate branch (Florova et al., 2015) or accelerate active to latent transition of PAI‐1 (Florova et al., 2018) resulted an in up to 8‐fold decrease in the MED of scuPA in treatment of TCN‐induced pleural injury. Previously, we hypothesized that DSP‐mediated PAI‐1‐TFT also increases the efficacy of fibrinolytic therapy (Florova et al., 2018). To test this hypothesis, we evaluated the effect of DSP, a small peptide molecule, that unlike anti‐PAI‐1 mAbs interacts with the enzyme, on the outcomes of fibrinolytic therapy with sctPA and scuPA in rabbit models of chemically induced and infectious pleural injury.

## 2 EXPERIMENTAL PROCEDURES

### 2.1 Proteins and reagents

Recombinant wild type human PAI‐1, tcuPA, tctPA, human fibrinogen, and FITC‐fibrinogen (3 moles of fluorescein per mole of fibrinogen) were from Molecular Innovations (Novi, MI). Chromogenic uPA and tPA substrate were from Centerchem Inc. (Norwalk, CT). DSP (EEIIMD) was synthesized by GenScript USA; (Piscataway, NJ). Prourokinase (scuPA) was a gift from LTI (TX) and sctPA (Alteplase) was from Genentech (CA). Fluorogenic plasmin substrate SN‐5, human Glu‐plasminogen, and plasmin standard were from Haematologic Technologies Inc. (HTI, Essex Junction, VT). Pleural fluid protein concentrations were determined using a BCA protein assay (Pierce, Rockford, IL). The 20–50 mM M Hepes/NaOH (pH 7.5) buffers with or without BSA (1 mg/ml) and/or NaCl (20 mM) were used to carry out all *in vitro* and *ex vivo* experiments.

### 2.2 Animal protocols

All animal procedures were approved by the Institutional Animal Care and Use Committee at The University of Texas Health Science Center at Tyler (IACUC protocols 499,616,617). Studies used female, pathogen‐free New Zealand white rabbits (3.0–3.6 kg; average age 18 weeks) from Charles River Laboratories (Wilmington, MA). These studies required a total of 107 rabbits, 43 for the model of TCN‐induced pleural injury and 64 for the model of empyema. A model of chemically induced pleural injury in rabbits was implemented as detailed previously (Idell et al., 2007). Briefly, a 3 ml mixture of TCN in ascorbic acid (20 mg/ml TCN (T3258, Sigma‐Aldrich), 2.5 mg/ml ascorbic acid (A7506, Sigma‐Aldrich) and lidocaine (1 mg/ml, UTHCT Compounding Pharmacy), was delivered by intrapleural injection. A model of acute empyema in rabbits was induced as described previously (Komissarov et al., 2016). Briefly, 1–5 × 108 cfu of *S*. *pneumoniae* (D39 strain, National Collection of Type Cultures, Salisbury UK) in 3 ml of 0.5% brain‐heart infusion agar (BD 238400, BD Diagnostic Systems) was delivered by intrapleural injection. Clavomox (10 mg/kg, subcutaneous, daily for 1–3 days as clinically indicated by Attending Veterinarian) (10000485, Zoetis) was started at 28–30 h post‐infection. Accumulation of pleural fluid and fibrin deposition was monitored using ultrasonography; detailed in the ultrasonography subsection. After 48 or 72 h for TCN‐induced pleural injury and empyema, respectively, intrapleural therapeutics were administered depending on treatment group via an 18‐gauge catheter, which was flushed with 0.5 ml PBS. In all experiments, samples of pleural fluids (0.5 ml) from each rabbit were collected prior to and at 10, 20, and 40 min after PAI‐1‐TFT and processed for later analysis (Florova et al., 2015; Karandashova et al., 2013; Komissarov et al., 2013, 2016). Anesthesia, postoperative pain medication, and animal care were provided as reported previously (Florova et al., 2015; Karandashova et al., 2013; Komissarov et al., 2013, 2016). with details provided in the supplement. Animals were monitored carefully for signs of distress, pain, and worsening clinical status. Euthanasia was accomplished using intravenous injection of 1 ml of commercial euthanasia solution (sodium pentobarbital 390 mg/ml and phenytoin 50 mg/ml) followed by exsanguination.

### 2.3 Ultrasonography

Development of pleural injury was monitored via ultrasonography. Briefly, B‐mode ultrasonography of the chest was performed (Komissarov et al., 2013, 2015) using a Logiq e system (GE Healthcare, Milwaukee, WI) using R5.2.x software and a multifrequency transducer model 12L‐RS at a working frequency of 10 MHz, as previously described (Komissarov et al., 2016).

### 2.4 Metrics of pleural injury

For each animal, gross lung injury scores (GLIS) were calculated as previously described (Florova et al., 2015; Karandashova et al., 2013; Komissarov et al., 2013). PAI‐1‐TFT was considered successful when GLIS ≤10. Multiple fibrin webs or sheets connecting the visceral and parietal pleura or “too numerous to count” (TNTC) strands and a fibrinous lung coating correlated with a GLIS = 50. Pleural thickening was measured using morphometry, as previously described (Komissarov et al., 2016).

### 2.5 Pharmacokinetic analysis

The residual DSP in pleural fluid samples was determined using LC MS/MS. Briefly, collected samples were extracted using acetonitrile, cleaned with Microcon centrifugal filters (MRCPRT010, MilliporeSigma) and injected via autosampler onto a Pinnacle DB C8 Column (9413332, Restek) reverse‐phase column operated at a flow rate of 200 μl/min. A gradient elution method using solvent A (99% H_2_O, 1% acetonitrile, 0.05% TFA) for 5 min, a gradient to 100% solvent B (1% H_2_O, 99% acetonitrile, 0.05% TFA) over 25 min, and 100% solvent B for 2 min was employed. Mass spectrometric analyses were performed on a TSQ Vintage (Thermo Fisher Scientific) mass spectrometer equipped with an electrospray ion source operating in positive mode and SRM detection mode. The apparent rate constant of intrapleural elimination of DSP (k_clr_) was estimated by fitting a single exponential equation to the plot of concentration of DSP in pleural fluids on time.

### 2.6 ELISA for quantitation of antigens and PAI‐1 activity

Commercial ELISA were used to assess the levels of total and active PAI‐1 (RBPAIKT‐TOT, RbPAIKT; Molecular Innovation Inc.), tissue necrosis factor‐α (TNF‐α; DY5670, R&D Systems), transforming growth factor‐β (TGF‐β; DY240‐05, R&D Systems), interleukin (IL)‐6 (DY7984, R&D Systems), IL‐8 (ELL‐IL8‐1, RayBiotech) in rabbit pleural fluids.

### 2.7 Measurements of uPA and tPA amidolytic and plasminogen activating activity

Amidolytic activity was measured in clear 96‐well flat bottom plates from Costar (Corning Inc.) using a Synergy™ HT Hybrid Reader (BioTek, Winooski, VT). Amidolytic activity was calculated from the initial slopes of the time traces of the change in absorbance at 405 nm upon hydrolysis of chromogenic as described previously (Komissarov et al., 2009, 2013). The plasminogen activating activity was determined from the relative rate of activation of exogenous Glu‐plasminogen added to the reaction mixture containing 0.1–0.5 µl of pleural fluid in 0.1 ml of Hepes buffer solution with BSA (1 mg/ml) as previously described (Florova et al., 2013, 2018). The reaction mixtures (0.1 ml in black 96‐well flat bottom plates from Costar) contained 100 nM of human Glu‐plasminogen with 0.2 mM of SN‐5 plasmin fluorogenic substrate (HTI, Essex Junction, VT). Plasmin activity was measured by recording changes in the fluorescence emission at 470 nm (F_470_) with time (excitation at 352 nm) using a Synergy™ HT Hybrid Reader (BioTek, Winooski, VT). The level of plasminogen activating activity [PA] was calculated from slopes of linear dependences of F_470_ from square of time (t^2^) (F_470_ = A [PA] t^2^ + B; where A and B are constants), and results of measurements of standards with known concentrations of tcuPA and tctPA as previously described (Komissarov et al., 2009, 2013).

### 2.8 Kinetics of intrapleural tPA and uPA inactivation and αM/uPA complex formation

The observed first order rate constants (k_obs_) for intrapleural inactivation of tPA or uPA and αM/uPA formation were determined from measurements of amidolytic activity with low molecular weight substrates, as previously described (Komissarov et al., 2013). The values of k_obs_ for intrapleural inactivation of tPA and uPA were estimated by fitting an exponential equation to the time dependences (10–40 min) of amidolytic activity. To determine intrapleural levels of free uPA and αM/uPA (possesses amidolytic activity against low molecular weight uPA substrates) (Komissarov et al., 2009, 2013) uPA activity was measured before (total uPA) and after (activity of uPA complexed with αM) supplementation of pleural fluid samples with 20–50 fold excess of recombinant rabbit PAI‐1, corresponding to the activities of αM/uPA and total uPA, respectively. Samples with known concentration of active uPA, tPA and αM/uPA were used as standards. To determine k_obs_ for uPA inactivation and αM/uPA accumulation, a single exponential equation was fit to the time‐dependences of the intrapleural concentrations of active free uPA ([free uPA] = [total uPA] − [αM/uPA]) or αM/uPA, respectively using SigmaPlot 14.0. The values of k_obs_ for intrapleural inactivation of tPA and uPA were also measured from time‐changes of plasminogen activating activity (Florova et al., 2015; Komissarov et al., 2013) and compared to the values obtained from measurements of amidolytic activity.

### 2.9 Fibrinolytic activity and fibrinolytic potential in pleural fluids

A Pilot Fibrinolytic Assay (FPA96F) was used to measure fibrinolytic potential (Beckert et al., 2019; Florova et al., 2015) and fibrinolytic activity (Florova et al., 2015, 2018; Komissarov et al., 2016) in pleural fluid samples. To prepare a FITC‐fibrin film, a 1:1 mixture of FITC‐tagged and unlabeled fibrinogen (0.4 mg/ml) in 0.05 M Hepes/NaOH (pH 7.5, 130 mM NaCl, 5 mM CaCl_2_) buffer at room temperature was transferred to black 96‐well flat bottom plates from Costar (Corning Inc, NY). Human thrombin (final concentration 10 nM) was added to each well and plates were dried overnight in a dark chemical hood at room temperature. 96‐well plates with FITC‐fibrin film were washed three times with 0.3 ml of cold Hepes/NaOH buffer and stored at −20°C up to one year. Fibrinolytic activity in pleural fluids was calculated from the slopes of time‐dependences of an increase in the fluorescence emission at 512 nm (excitation at 490 nm) due to the degradation of FITC‐fibrin. Fibrinolytic potential was determined as the difference between fibrinolytic activity in samples of pleural fluid at baseline (BL) or 24 h measured with and without supplementation with sctPA (Florova et al., 2015). To determine whether PAI‐1‐TFT affects fibrinolytic potential in rabbits, BL values were plotted against fibrinolytic potential at 24 h and the linear correlation was determined using Sigmaplot 14.0.

### 2.10 Statistics

Statistical significance was determined using the Kruskal–Wallis test as well as pairwise multiple‐comparison procedures (Dunn's multiple comparison test). Correlation coefficients (*r*) were calculated using the fit of the curve and used as a parameter to determine the best fit. Data were analyzed using SigmaPlot 12.3 for Windows (SPSS Inc.) and GraphPad Prism 8.2.1, as previously described (Florova et al., 2015; Komissarov et al., 2013, 2016).

## 3 RESULTS

### 3.1 Developing and treating chemically induced and infectious pleural injury in rabbits

**FIGURE 1:**
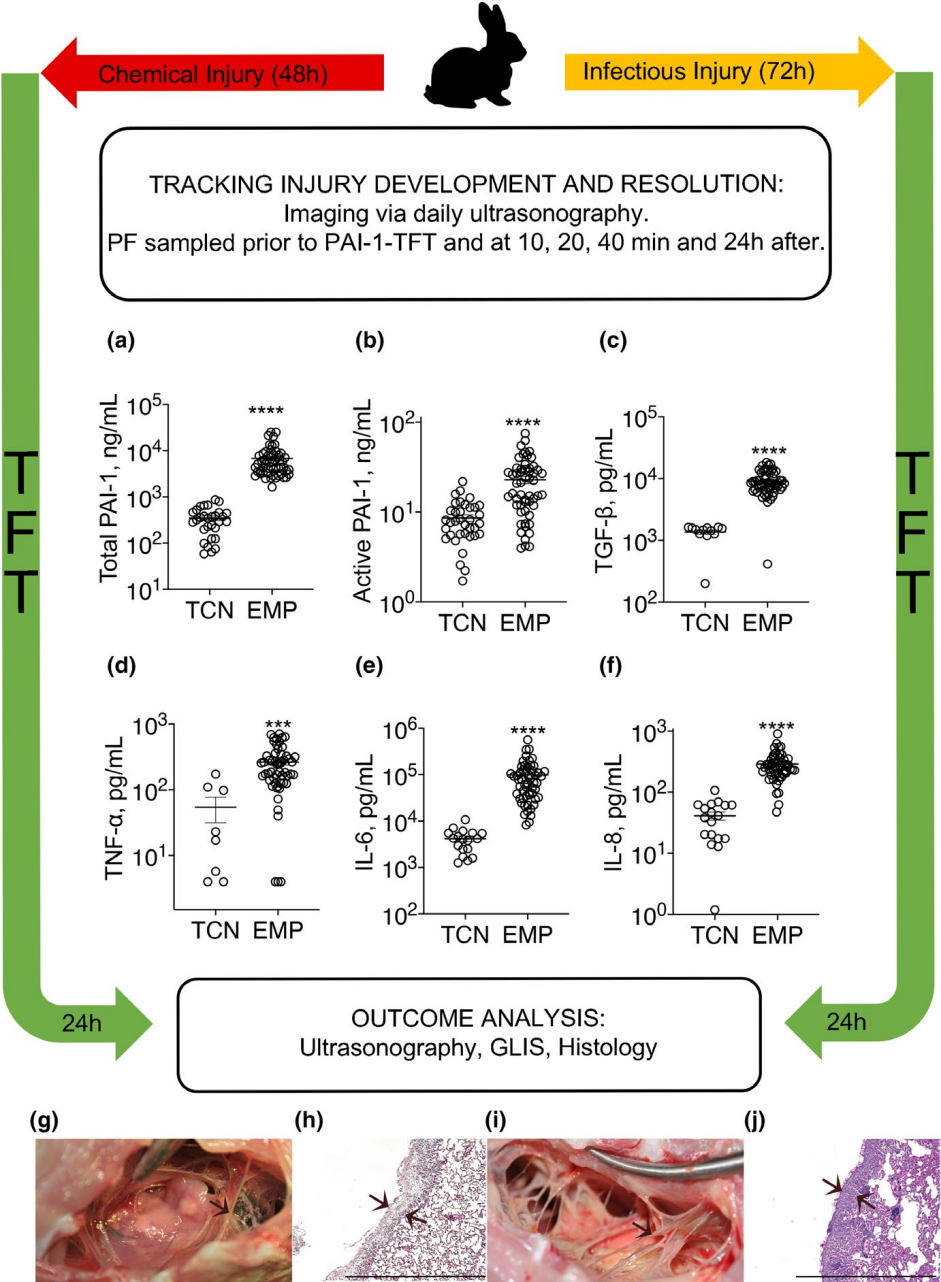
Models of chemically induced (TCN) and infectious (EMP) pleural injury in rabbits were used to test PAI‐1‐TFT. Time course of initiation, development, of TCN‐ (left; 0–48 h) and *S. pneumoniae* (right; 0–72 h) induced pleural injury in rabbits followed by treatment (TFT), and data analysis. Pleural injury was initiated by intrapleural injection of either TCN (Idell et al., 2007) or *S. pneumoniae* (Komissarov et al., 2016). Injury progression was tracked using ultrasonography. Intrapleural levels of PAI‐1, total (a; *n* = 30 and 55) and active (b; *n* = 36 and 55); transforming growth factor‐ß (TGF‐β); (c; *n* = 12 and 55); tissuenecrosis factor‐α (TNF‐α) (d; *n* = 8 and 55); interleukin (IL)‐6 (e; *n* = 18 and 55); IL‐8 (f; *n* = 18 and 53), were determined in baseline samples of pleural fluid (PF) collected from animals with chemically‐induced (TCN) and infectious (EMP) pleural injury, respectively. Data are presented as scatter plots, showing mean with SEM, *p* < 0.01 (**), *p* < 0.0001 (****) using a two‐tailed Kolmogorov‐Smirnov test. PAI‐1‐TFT was delivered at 48 (chemically induced) and 72 (infectious) hours and outcomes were assessed 24 h later using ultrasonography (data not shown), postmortem visualization of the pleural space during necropsy, and by hematoxylin and eosin (H&E) of tissue samples. A Gross Lung Injury Score (GLIS) (Florova et al., 2015; Komissarov et al., 2016) was used for visual assessment; GLIS ranges from 0 to 50, where 0 refers to a clear pleural space and 50 to fibrin formations that are too numerous to count; GLIS ≤10 considered successful PAI‐1‐TFT. Representative photographic images of GLIS = 50 (arrows indicate fibrin adhesions) for TCN‐induced (g) and infectious (i) pleural injury after treatment with a vehicle control. Representative images of H&E staining of paraffin‐embedded lung tissue demonstrate pleural thickening in TCN‐induced (h) and infectious (j) injury. Arrows indicate the pleural surface and the basement membrane of the pleura. There was no statistically significant difference (*p* > 0.05) in pleural thickening between models. Images were obtained at 4x on a Cytation 5 (Biotek) with 5–6 rabbits/group; scale bars, 1 mm

Two validated models of pleural injury (chemically induced and infectious, Figure 1) (Florova et al., 2015, 2018; Idell et al., 2007; Karandashova et al., 2013; Komissarov et al., 2013, 2015, 2016) were used to evaluate the effect of DSP on the outcomes of fibrinolytic therapy. Pleural injury was monitored and verified using ultrasonography. Levels of PAI‐1 (active and total), TGF‐β, TNF‐α, IL‐6 and IL‐8 (Figure 1a–f) were measured in samples of pleural fluid collected immediately prior to PAI‐1‐TFT (at 48 or 72 h for the chemically induced and infectious injuries, respectively). Notably, while levels of PAI‐1, TGF‐β, TNF‐α, IL‐6, and IL‐8 were elevated in both models, they were higher (*p* < 0.05) in empyema (Figure 1a–f). Both models featured robust intrapleural fibrin deposition detected by ultrasonography (data not shown) and documented by photography during necropsy (Figure 1g,i). The highest Gross Lung Injury Score (GLIS=50), which corresponds to fibrin strands, nets, and aggregates that were too numerous to count (TNTC), was the same for both models (Figure 1g,i). While infectious pleural injury caused greater fibrin deposition on visual inspection (Figure 1i), there was no statistically significant difference in the pleural thickening (Figure 1h,j). Thus, using DSP for PAI‐1‐TFT in models of pleural injury that feature different levels of PAI‐1 and inflammatory responses (Figure 1), allows us to validate the approach, assess the effects of increases in the molecular target on doses of plasminogen activator and DSP, and test the concept that precision PAI‐1‐TFT improves therapeutic outcomes.

### 3.2 DSP improves therapeutic outcomes of ineffective doses of plasminogen activators in both chemically induced and infectious pleural injury in rabbits

**FIGURE 2:**
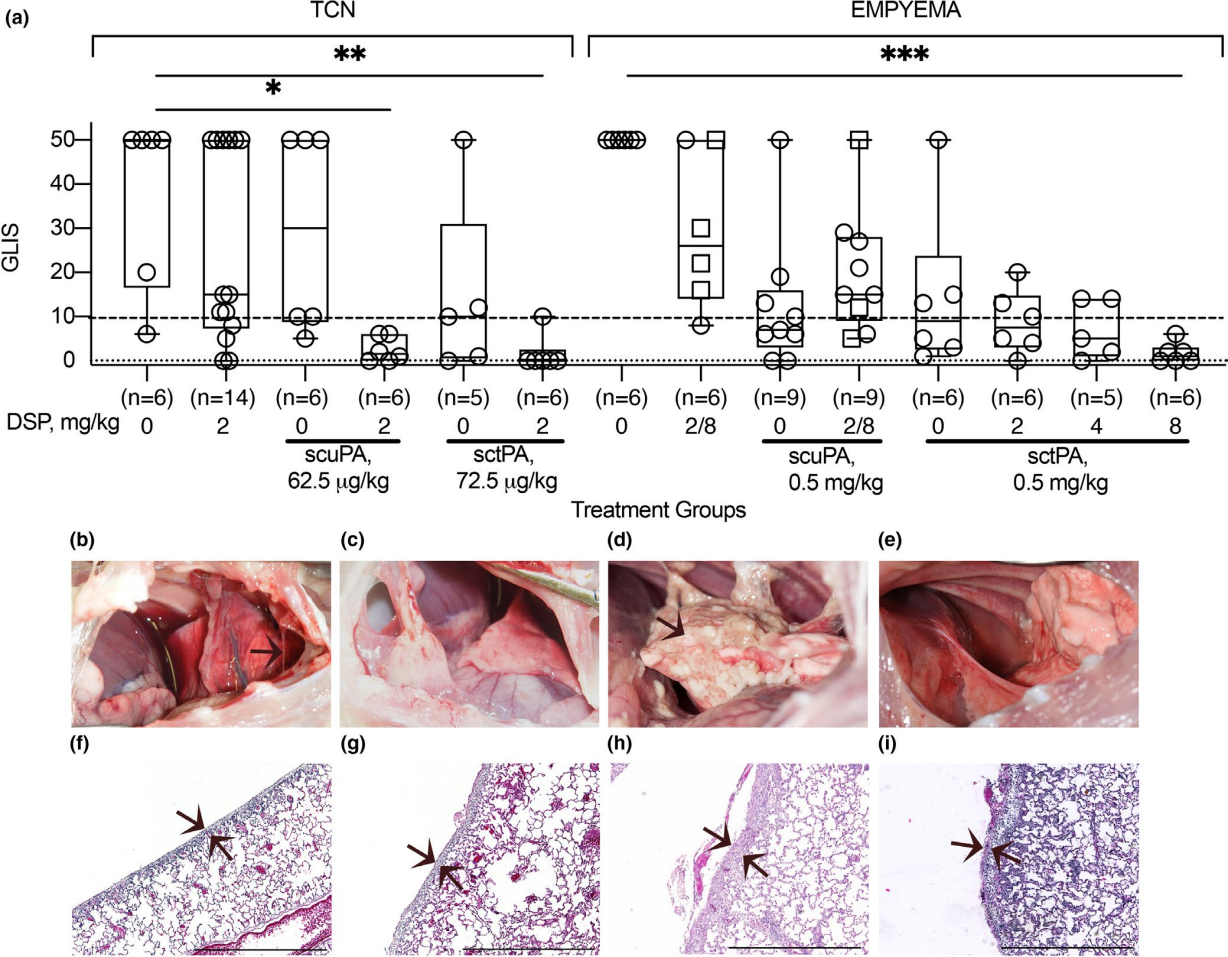
PAI‐1‐TFT results in a decrease in the dose of plasminogen activator in chemically induced (TCN) and infectious (empyema) pleural injury. The efficacy of fibrinolytic therapy was measured by GLIS (GLIS ≤ 10, at or below the dashed line, indicates successful PAI‐1‐TFT). (a; TCN) Animals were treated (from left to right) with: vehicle control (*n* = 6); 2.0 mg/kg DSP alone (*n* = 14); an ineffective dose (62.5 µg/kg) of scuPA (*n* = 6); 62.5 µg/kg of scuPA with 2.0 mg/kg DSP (*n* = 6); an ineffective dose (72.5 µg/kg) of sctPA (*n* = 5); 72.5 µg/kg of sctPA with 2.0 mg/kg DSP (*n* = 6); (a; empyema) Animals were treated (from left to right) with: vehicle control (*n* = 6); 2.0 (*n* = 2) or 8.0 (*n* = 4; squares) mg/kg DSP alone; ineffective dose of scuPA (0.5 mg/kg; *n* = 9); 0.5 mg/kg of scuPA with 2.0 mg/kg (*n* = 6) or 8.0 mg/kg (*n* = 3; squares) DSP; ineffective dose (0.5 mg/kg) of sctPA alone (*n* = 6) and with 2.0 (*n* = 6), 4.0 (*n* = 5), and 8.0 mg/kg (*n* = 6) DSP. Statistically significant differences determined using Kruskal–Wallis test; *, ** and *** denote *p* < 0.05; <0.01 and <0.001, respectively. Representative photographic images of pleural spaces of animals with TCN‐induced pleural injury successfully (GLIS = 0) treated with 2.0 mg/kg DSP with 62.5 µg/kg of scuPA (b); or 72.5 µg/kg of sctPA (c) and animals with infectious pleural injury treated with 8.0 mg/kg DSP with 0.5 mg/kg scuPA (GLIS = 50; d) or with 0.5 mg/kg sctPA (GLIS = 0; e). Representative images of H&E staining of paraffin embedded lung tissue from the same animals (f–i). While animals with TCN‐induced injury (f,g) demonstrate a statistically significant decrease in pleural thickening (*p* < 0.01) compared to vehicle treated controls (Figure 1), animals with infectious pleural injury (h,i) did not (*p* > 0.05). Statistical significance was determined using an unpaired, 2‐tailed Kolmogorov‐Smirnov test. Scale bars, 1 mm. Images were obtained at 4× on a Cytation5 (Biotek) with 5–6 rabbits/group

**FIGURE 3:**
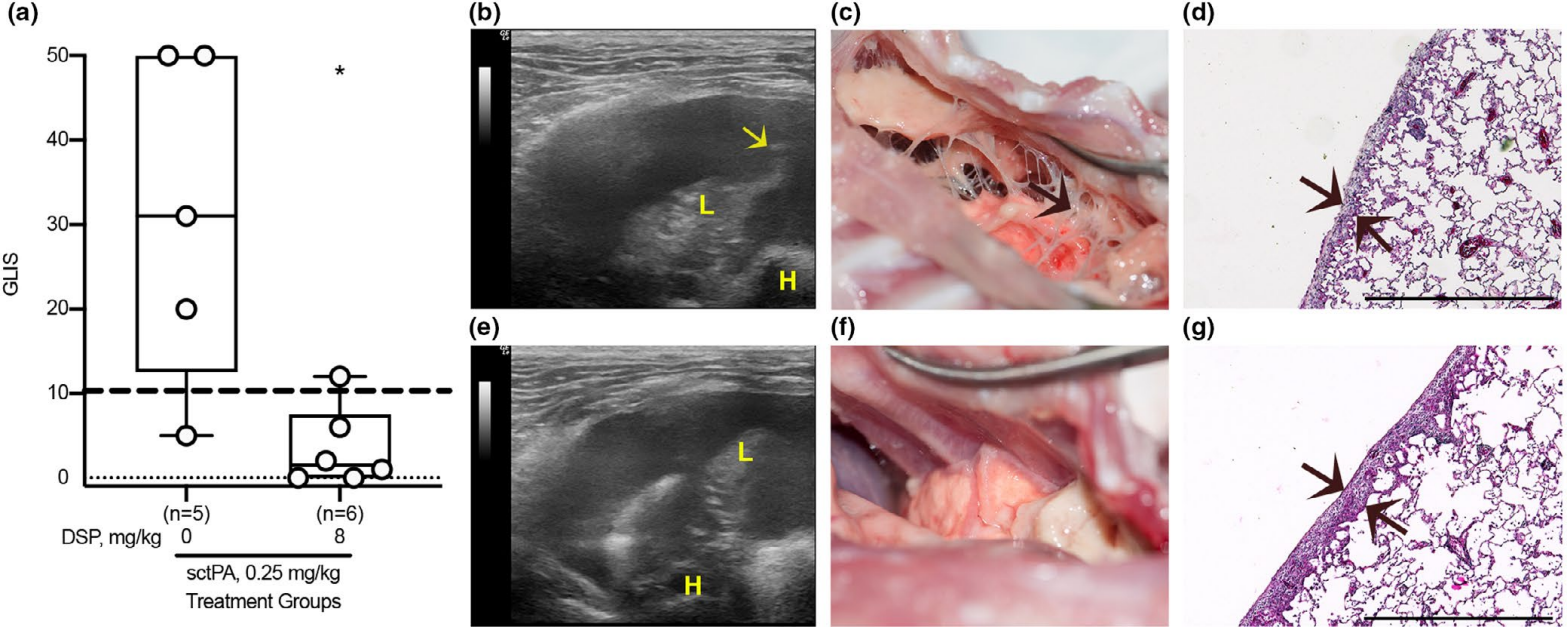
DSP (8.0 mg/kg) increases the efficacy of intrapleural fibrinolytic therapy by 8‐fold and converts an ineffective dose of sctPA (0.25 mg/kg) into a MED for treatment of acute empyema in rabbits. The effect of DSP on the efficacy of fibrinolytic therapy of infectious pleural injury with an ineffective dose of sctPA (0.25 mg/kg), measured by GLIS (GLIS ≤ 10 at or below the dashed line indicates successful PAI‐1‐TFT (a). Animals with acute empyema were treated (from left to right) with sctPA (0.25 mg/kg) alone (*n* = 5) or together with 8.0 mg/kg DSP (*n* = 6). The data are shown as a box plot; * denotes a statistically significant (*p* < 0.05) difference determined using an unpaired, two‐tailed Kolmogorov‐Smirnov test. Representative ultrasonography images of the pleural space 24 h after treatment with 0.25 sctPA alone (b), and in combination with 8.0 mg/kg DSP (e) show an acoustic window for lung (L), heart (H), and a fibrin strand (arrow) in the pleural space. Photographs of the pleural space 24 h after unsuccessful (GLIS = 50; arrow indicates fibrin adhesions) treatment with sctPA alone (c), and successful fibrinolytic therapy (GLIS = 5) with sctPA and DSP (f). H&E staining of paraffin embedded lung tissue of animals treated with sctPA alone (d) and together with DSP (g). Arrows indicate the pleural surfaces and basement membranes of the pleura. Scale bars: 1 mm. Images were obtained at 4x on a Cytation 5 (Biotek) with 5 rabbits/group

The treatment and outcomes analyses of the two models of pleural injury are shown in Figure 1. First, DSP alone was used to treat pleural injury in both models to determine its effects on the endogenous fibrinolytic system. The results (Figure 2) clearly indicate that even a relatively high dose of DSP (2.0 mg/kg) is insufficient to mobilize endogenous fibrinolysis effectively once pleural injury has developed; there was no reversal effect with DSP alone. DSP alone, even at a 4‐fold higher dose (8.0 mg/kg), was also ineffective (GLIS>10) in treatment of empyema (Figure 2). Next, PAI‐1‐TFT with a bolus injection of sctPA or scuPA in combination with DSP was delivered at 48 h (Figure 2a; TCN) and at 72 h (Figure 2a; Empyema) after initiation of pleural injury (Figure 1). Control animals were treated with plasminogen activator alone (Figure 2). Aliquots of pleural fluid were withdrawn prior to treatment and at 10, 20, 40 min and at 24 h after PAI‐1‐TFT. Outcomes were assessed 24 h after treatment using ultrasonography and morphometric GLIS scores determined at necropsy. PAI‐1‐TFT was considered successful if GLIS≤10, as we previously reported (Florova et al., 2015; Karandashova et al., 2013). To test the effect of DSP on the outcome in TCN‐induced pleural injury, ineffective (GLIS>10) doses of scuPA and sctPA were selected. The dose of scuPA (62.5 µg/kg; 1/8 of the MED for scuPA alone (Idell et al., 2007) previously shown to be effective when used with anti‐PAI‐1 mAbs for treatment of TCN‐induced injury (Florova et al., 2015, 2018), and an equimolar dose of sctPA (72.5 µg/kg; 1/2 of the MED for sctPA alone) (Idell et al., 2007). The results of DSP‐mediated PAI‐1‐TFT in chemically induced pleural injury are shown in Figure 2a, TCN. DSP (2.0 mg/kg) renders otherwise ineffective doses of both scuPA and sctPA effective (GLIS≤10; Figure 2b,c). Notably, PAI‐1‐TFT caused a statistically significant (*p* < 0.01) decrease in pleural thickening (Figure 2f,g versus Figure 1h). Targeting exosite interactions between PAI‐1 and plasminogen activator using DSP resulted in a 2‐ and 8‐fold increase in the efficacy of fibrinolytic therapy with sctPA and scuPA, respectively (Figure 2a, TCN). *S*. *pneumoniae* induced pleural injury results in higher levels of the molecular target, PAI‐1, compared to TCN‐induced injury (Figure 1a,b). Moreover, MEDs of sctPA (0.145 mg/kg) and scuPA (0.5 mg/kg) that effectively treat of chemically induced injury were ineffective in empyema (Komissarov et al., 2016). The increase in intrapleural PAI‐1 in infectious injury (Figure 1) correlates with an increase in the MED to 2.0 mg/kg (Idell et al., 2007) for both sctPA and scuPA. A dose of 0.5 mg/kg, ineffective for both sctPA (n=6) and scuPA (*n* = 6) (Figure 2a, Empyema), was selected to test the outcomes of DSP‐mediated PAI‐1‐TFT. First, 2.0 mg/kg DSP, a dose that was effective in chemically induced pleural injury (Figure 2a, TCN) in combination with 0.5 mg/kg of sctPA or scuPA, was tested. In contrast to the treatment of chemically induced injury (Figure 2a, TCN), 2.0 mg/kg DSP adversely affected the outcome of fibrinolytic therapy with scuPA but showed a trend (*p*>0.05) towards improvement with sctPA (Figure 2a, Empyema). An increase in the level of the molecular target, PAI‐1, that results in a higher MED for plasminogen activator in the model of infectious pleural injury suggests that more DSP is needed to improve the outcome of PAI‐1‐TFT. This hypothesis was tested by sequential two‐fold escalations of the DSP dose in combination with sctPA (0.5 mg/kg) (Figure 2a, Empyema). An increase in the dose of DSP from 2.0 to 4.0 (*n* = 5) and 8.0 mg/kg (*n* = 6) (Figure 2a, Empyema) led to effective (GLIS≤10) PAI‐1‐TFT with sctPA (Figure 2e). There was no statistically significant decrease in pleural thickening after successful PAI‐1‐TFT (Figure 2i) compared to vehicle controls (Figure 1j). These results show that an increase in the level of the molecular target in empyema correlates with an increase in the MED of sctPA and a corresponding increase in the dose of DSP needed for effective PAI‐1‐TFT. In contrast to sctPA, combining DSP (2.0 and 8.0 mg/kg) with scuPA (0.5 mg/kg) was less effective than scuPA alone (Figure 2a, Empyema), resulting in GLIS>10 and robust pleural thickening (Figure 2d,h). Thus, DSP, even at a high dose such as 8.0 mg/kg, affects tPA and uPA induced intrapleural fibrinolysis differently in our model of infectious pleural injury in rabbits. Next, we determined whether the MED of sctPA in empyema treatment could be further reduced when administered with 8.0 mg/kg DSP. Animals with acute empyema were treated with sctPA (0.25 mg/kg) alone (*n* = 6) and in combination with 8.0 (*n* = 6) mg/kg DSP (Figure 3). Clearance of fibrin from the pleural space was visualized by ultrasonography (Figure 3b,e) and confirmed using photography (Figure 3c,f). DSP (8.0 mg/kg) dramatically improves the outcome of PAI‐1‐TFT (*p* < 0.05), converting an ineffective dose of sctPA 0.25 mg/kg to a MED (Figure 3a,c,f) that is 8‐fold less than the MED of sctPA alone (2.0 mg/kg) (Komissarov et al., 2016). There was no statistically significant difference in pleural thickness between successful (Figure 3g) and unsuccessful (Figures 1j and 3d) treatments.

### 3.3 Pharmacokinetics of DSP in the rabbit pleural space

**FIGURE 4:**
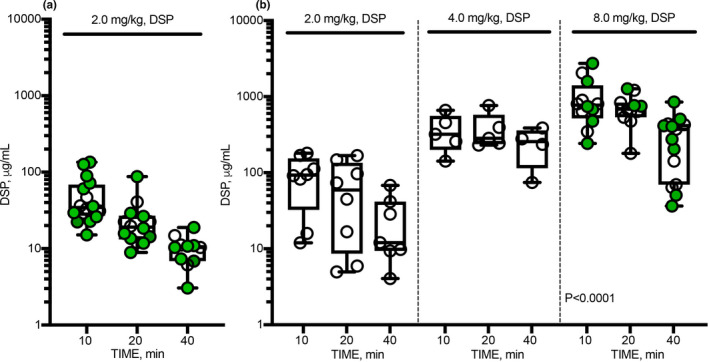
Pharmacokinetics of DSP in pleural fluid of animals with chemically induced (a) and infectious (b) pleural injury. LC MS/MS analyses were used to measure the levels of DSP in pleural fluids of rabbits at 10, 20 and 40 min after PAI‐1‐TFT. Animals with chemically‐induced pleural injury (a) were treated successfully (GLIS ≤ 10; green circles) with DSP (2.0 mg/kg) in combination with either 72.5 µg/kg (*n* = 6) of sctPA or 62.5 µg/kg (*n* = 5) of scuPA; or unsuccessfully (GLIS > 10; empty circles) with DSP alone (*n* = 6); LC MS/MS analyses of pleural fluids from animals with *S. pneumoniae* induced pleural injury (b) treated unsuccessfully (GLIS > 10; empty circles) with sctPA (0.5 mg/kg) in combination with (from left to right): 2.0 (*n* = 6) and 4.0 (*n* = 6) mg/kg DSP. Levels of DSP in pleural fluids of animals treated successfully (GLIS ≤ 10; green circles) with 8.0 mg/kg DSP in combination with 0.5 (*n* = 6) and 0.25 (*n* = 6) mg/kg sctPA are shown in the rightmost panel. The results from unsuccessful (GLIS > 10; empty circles) treatments of infectious pleural injury with DSP alone with doses of 2.0 (*n* = 2) and 8.0 (*n* = 4) mg/kg are added to the corresponding panels. The data are presented as box and whiskers plots, showing all points. The values of the observed first order rate constants for intrapleural DSP clearance (k_clr_), determined by fitting a single exponential equation to the data were 0.05 ± 0.01, 0.06 ± 0.01 and 0.07 ± 0.02 min^−1^ (*p* < 0.05, *r* > 0.85) for treatment of TCN‐induced injury with 2.0 mg/kg DSP alone or with 62.5 µg/kg of scuPA or with 72.5 µg/kg of sctPA, respectively (a), and 0.04 ± 0.02, 0.03 ± 0.02, 0.05 ± 0.03, 0.04 ± 0.01, and 0.04 ± 0.02 min^−1^ (*p* < 0.05, *r* > 0.6) for treatments with 0.5 mg/kg of sctPA in combination with 2.0, 4.0 and 8.0 mg/kg DSP, 8.0 mg/kg DSP alone and in combination with 0.25 mg/kg of sctPA, respectively (b

The results of Liquid Chromatography in tandem with Mass Spectrometry (LC MS/MS) analyses demonstrated an increase in the level of DSP in pleural fluid as the dose of the peptide was increased from 2.0 to 8.0 mg/kg (Figure 4). The intrapleural level of DSP decreases sharply in the first 10 min after injection (data not shown), followed by a slower phase with apparent first order rate constants of DSP clearance (k_clr_) ranging at 0.03–0.07 min^−1^ in both models. Pharmacokinetic analyses of DSP in pleural fluid of animals with TCN‐induced pleural injury (Figure 4a) and empyema (Figure 4b) confirm the presence of DSP for over 40 min after treatment as well as an increase in the intrapleural level of DSP with dose escalation (Figure 4b). The highest k_clr_ were observed in the model of TCN‐induced injury (0.05±0.01, 0.06±0.01, and 0.07±0.02 min^−1^ for 2.0 mg/kg DSP alone and together with sctPA or scuPA, respectively). The rate of DSP elimination becomes slower (*p* < 0.05) in rabbit model of empyema (k_clr_ were 0.04±0.02 and 0.05±0.02 min^−1^, for 2.0 mg/kg DSP with sctPA or scuPA, respectively). Increasing the dose of DSP to 4.0 and 8.0 mg/kg did not affect dramatically rate of the slow phase of DSP clearance. The values of k_clr_ for treatments with 0.5 mg/kg of sctPA with 4.0 and 8.0 mg/kg DSP and 0.25 mg/kg of sctPA with 8.0 mg/kg DSP were 0.03±0.02, 0.05±0.03 and 0.04±0.02 min^−1^, respectively. The k_clr_ for treatment with 8.0 mg/kg DSP alone (0.04±0.01 min^−1^) was similar to those observed with plasminogen activators. Thus, fibrinolytic therapy does not affect the rate of elimination of DSP from the pleural space in either model of pleural injury. The average intrapleural concentration of DSP 40 min after treatment with 2.0 mg/kg increases from 10 µg/mL in TCN‐induced injury to 36 µg/mL in empyema and increases further to >300 µg/mL after a dose escalation to 8.0 mg/kg DSP, with which PAI‐1‐TFT is successful (Figure 4; green symbols). These results indicate clearly that an increase in the efficacy of PAI‐1‐TFT in infectious pleural injury with otherwise ineffective doses of sctPA correlates with an increase in the intrapleural level of the peptide. Furthermore, these results support the hypothesis that an increase in the level of the molecular target, PAI‐1, observed in empyema model (Figure 1) necessitates an increase in both the MED of sctPA and DSP to make PAI‐1‐TFT successful.

### 3.4 Changes in intrapleural plasminogen activating and fibrinolytic activities during PAI‐1‐TFT

**FIGURE 5:**
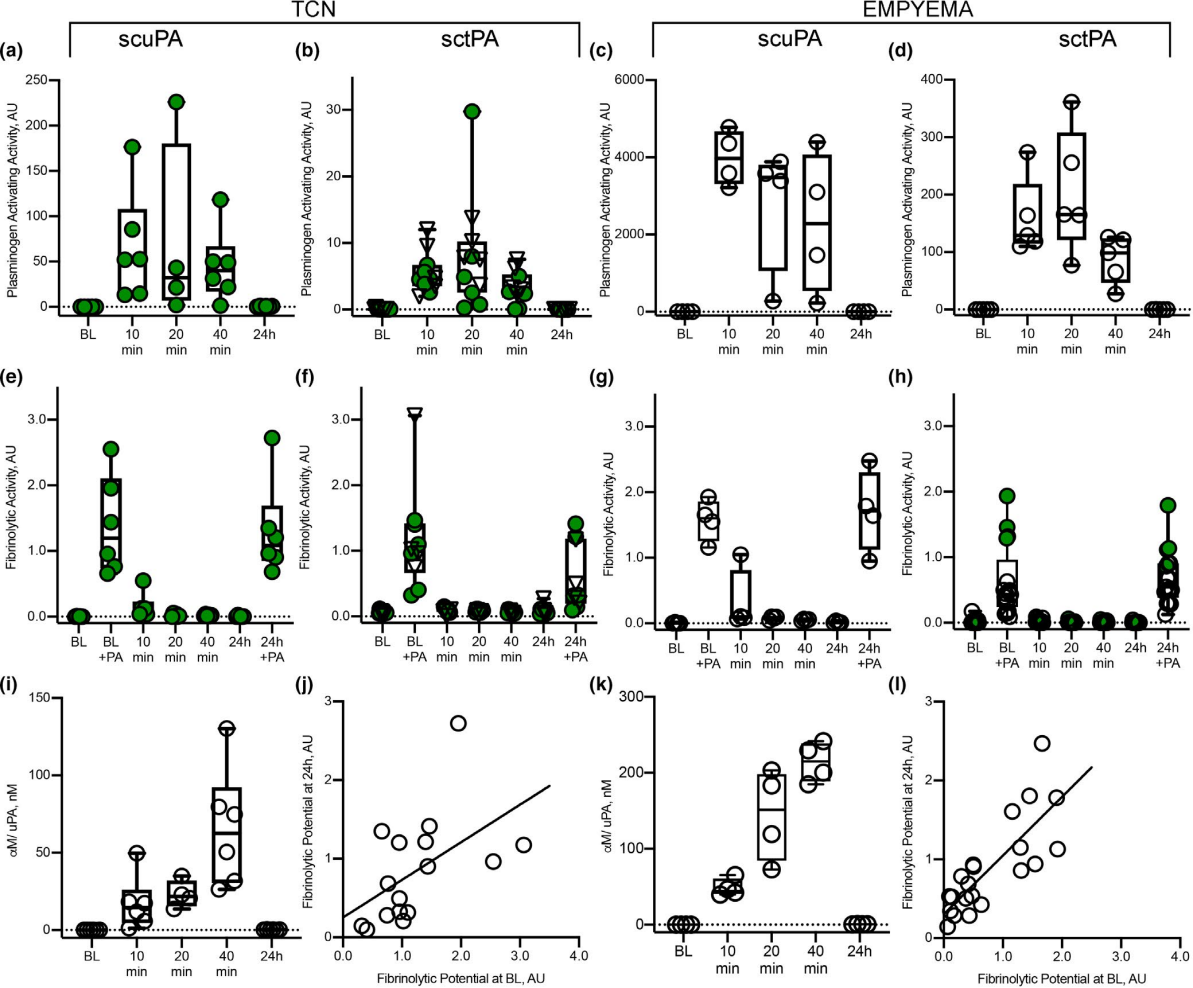
Changes in the plasminogen activating (a–d) and fibrinolytic (e–h) activities, levels of αM/uPA complexes (i,k), and correlation between fibrinolytic potential measured at the baseline and at 24 h after PAI‐1‐TFT in pleural fluids of rabbits following chemically induced (a, b, e, f, i, j) and infectious (c, d, g, h, k, l) pleural injury. Plasminogen activating and fibrinolytic activity was measured in samples of pleural fluid collected at baseline (BL); 10, 20, 40 min and at 24 h after PAI‐1‐TFT with DSP 2.0 mg/kg in combination with 62.5 µg/kg of scuPA (*n* = 6; a, e) or 72.5 µg/kg of sctPA (*n* = 6; b, f) (chemically induced injury; TCN); 0.5 mg/kg scuPA (*n* = 4; c, g) (infectious injury; empyema). Green and empty circles represent successful (GLIS ≤ 10) and unsuccessful (GLIS > 10) PAI‐1‐TFT, respectively. Reversed triangles (b, f) represent treatment with sctPA alone (*n* = 5, GLIS > 10). D represents treatment with 0.5 mg/kg sctPA with 2.0 mg/kg DSP (*n* = 6); h, treatments (*n* = 17) with 0.5 mg/kg sctPA in combination with 2.0 (*n* = 6), 4.0 (*n* = 5) and 8.0 (*n* = 4) mg/kg DSP and 0.25 mg/kg sctPA with 4.0 mg/kg DSP (*n* = 2). Fibrinolytic activity in aliquots of pleural fluids withdrawn prior to (BL) and 24 h after PAI‐1‐TFT were analyzed with (+PA) or without supplementation with 4.0 nM of plasminogen activator (Florova et al., 2015). Levels of αM/uPA in pleural fluids at BL, 10, 20, 40 min and 24 h (i, k) were measured as described previously (Komissarov et al., 2013) and plotted against time. Fibrinolytic potential (Florova et al., 2015) was determined in samples of pleural fluid withdrawn at 24 h and plotted versus corresponding values determined for baseline (BL) for TCN‐induced injury (*n* = 16; j) and empyema (*n* = 21, l). The solid lines represent the best fit (*p* < 0.05) of a linear equation to the data with slopes 0.48 ± 0.20 and 0.74 ± 0.13, and *r* = 0.54 and 0.80 for TCN‐induced and infectious pleural injury, respectively

**FIGURE 6:**
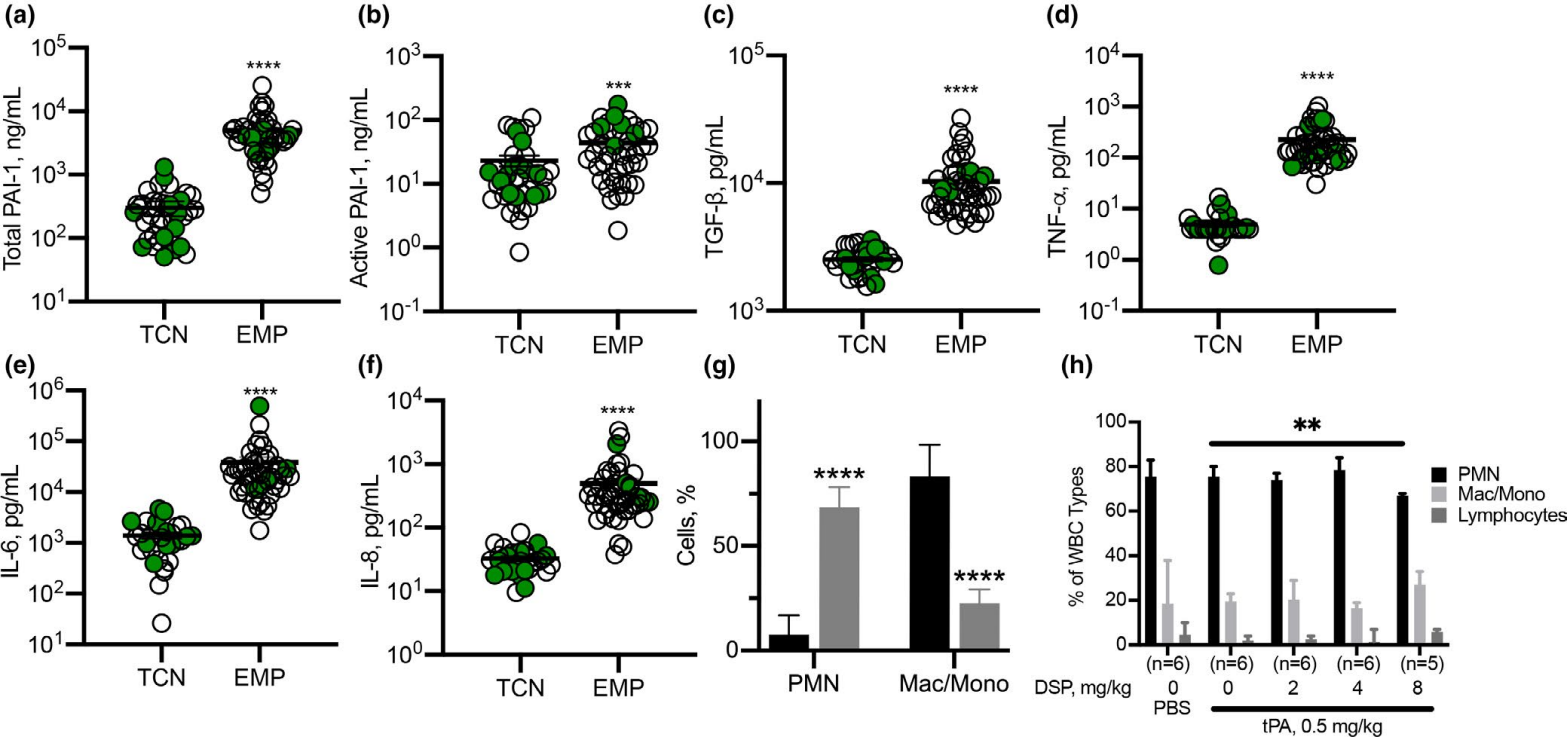
Inflammatory parameters were higher in rabbits with *S. pneumoniae*‐induced pleural injury 24 h after PAI‐1‐TFT. Pleural fluid from rabbits with TCN‐induced injury or *S. pneumoniae*‐induced pleural injury (EMP) were subjected to ELISA to measure: total PAI‐1 (a), active PAI‐1 (b), transforming growth factor‐ß (TGF‐β); (c), tissue necrosis factor‐α (TNF‐α); (d), interleukin (IL)‐6 (e), and IL‐8 (f), 24 h after PAI‐1‐TFT. Significantly elevated levels of all targets were detected in pleural fluids of rabbits with empyema. There was no statistically significant difference between different treatments of each type of pleural injury. Data are presented as the mean ± SEM. Significance was determined using an unpaired, two‐tailed Kolmogorov‐Smirnov test. ****p < 0.0001 (total PAI‐1 *n* = 41, *n* = 45; active PAI‐1; *n* = 37, *n* = 44; TGF‐β, *n* = 31, *n* = 47; TNF‐α, *n* = 31, *n* = 50; IL‐6, *n* = 31, *n* = 51; IL‐8, *n* = 32, *n* = 49 for TCN‐induced injury and empyema, respectively). White blood cells (WBC) differential counts in pleural fluids from rabbits with TCN (black) ‐ and *S. pneumoniae*‐(grey) induced pleural injury (d). Pleural fluids were collected at necropsy. Neutrophil predominance was noted in *S. pneumoniae*‐induced injury while a mixed myeloid cell distribution was observed in chemically induced injury. Results of a Mann–Whitney test on ranks showed a statistically significant change (*p* < 0.0001) in cell populations. Data are presented as a grouped vertical bar graph with standard deviation. Pleural fluid WBC differentials in *S. pneumoniae*‐induced PAI‐1‐TFT treated pleural injury in rabbits (h). A significant decrease (*p* < 0.01 (**)) in the number of PMN is noted between rabbits treated with 0.5 mg/kg sctPA and 0.5 mg/kg sctPA with 8.0 mg/kg DSP. Significance was determined using an unpaired, two‐tailed Kolmogorov‐Smirnov test. Data are presented as a grouped vertical bar graph with standard deviation

**FIGURE 7:**
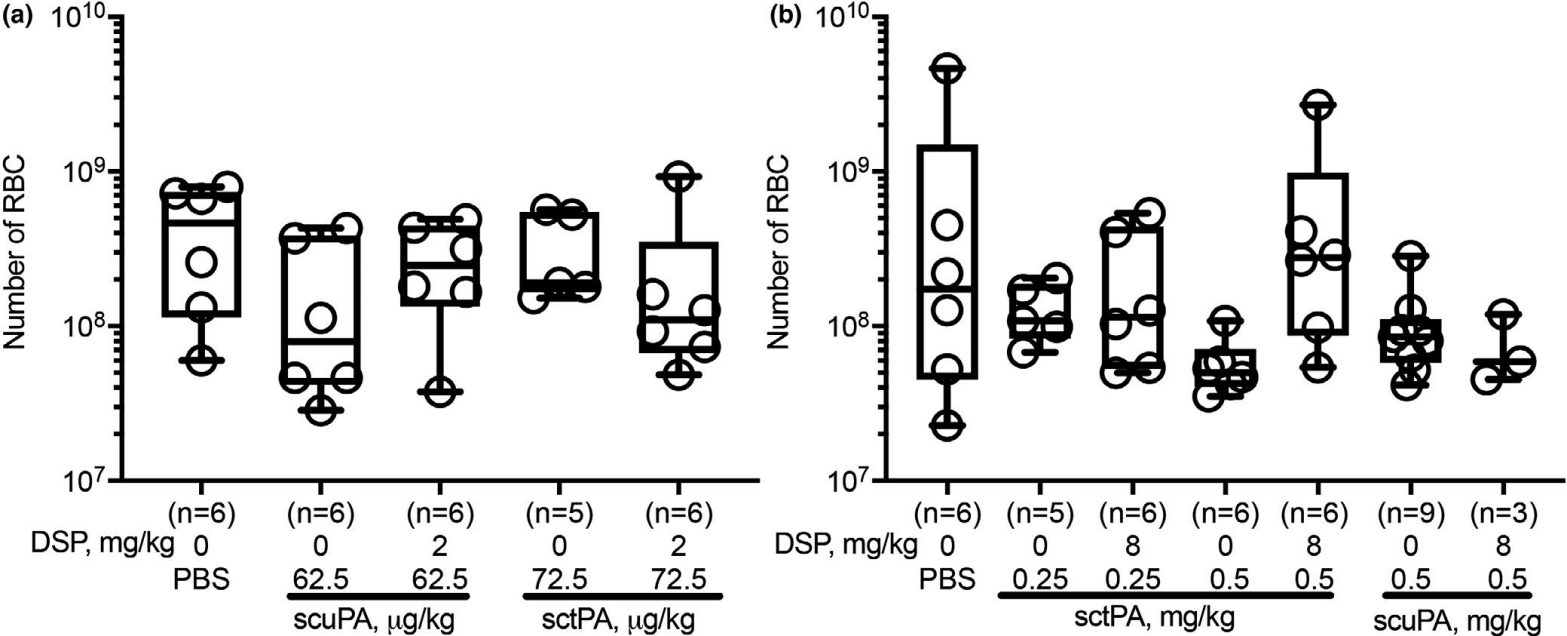
Low dose PAI‐1‐TFT induces no intrapleural bleeding. Pleural fluids from rabbits with TCN‐induced (a) or *S. pneumoniae*‐induced (b) pleural injury were collected at necropsy and red blood cells (RBC​) were counted using a Beckman Coulter. (a) Animals with TCN‐induced pleural injury were treated (from left to right) with vehicle control (*n* = 6); 62.5 µg/kg of scuPA alone (*n* = 6); and with 2.0 mg/kg DSP (*n* = 6); 72.5 µg/kg of sctPA alone (*n* = 5), and with 2.0 mg/kg DSP (*n* = 6). (b) Animals with infectious pleural injury were treated (from left to right) with vehicle control (*n* = 6); 0.25 mg/kg of sctPA alone (*n* = 5), and with 8.0 mg/kg DSP (*n* = 6), 0.5 mg/kg of sctPA alone (*n* = 6), and with 8.0 mg/kg DSP (*n* = 6), 0.5 mg/kg of scuPA alone (*n* = 9), and with 8.0 mg/kg DSP (*n* = 3). No statistically significant differences in RBCs were observed in either model after treatment with PAI‐1‐TFT as compared to vehicle treated animals. Data are presented as box and whisker plots (all points shown)

Intrapleural plasminogen activating (Figure 5a–d) and fibrinolytic (Figure 5e–h) activities were measured in pleural fluids prior to (baseline) and at 10, 20 and 40 min and at 24 h after treatment, as described previously (Florova et al., 2015, 2018; Komissarov et al., 2013, 2016). There was no detectable plasminogen activating activity at baseline or at 24 h (Figure 5a–d). The fast phase of intrapleural inhibition of plasminogen activators, which occurs within the first 10 min after treatment (Komissarov et al., 2013), was followed by a slower phase (Figure 5a–d). The observed rate constants (k_obs_) of intrapleural inactivation during PAI‐1‐TFT of chemically induced pleural injury, were in the range of 0.02– 0.05 min^−1^ for tPA and uPA plasminogen activating activity. The values of k_obs_ for intrapleural inactivation of tPA (with 2.0–8.0 mg/kg DSP) and uPA (with 2.0 mg/kg DSP) during PAI‐1‐TFT of infectious pleural injury were 0.024±0.010 and 0.014±0.008 min^−1^, respectively. Fibrinolytic activity was suppressed at baseline and at 24 h after treatment (Figure 5e–h). Supplementing pleural fluids collected at baseline and 24 h with plasminogen activator (which simulates fibrinolytic therapy) resulted in a significant increase in the fibrinolytic activity (Figure 5e–h). However, over the first 10 min after PAI‐1‐TFT, fibrinolytic activity in pleural fluids sharply decreases until it reaches almost steady state level (Figure 5e–h), which is less than 10% of that in supplemented baseline. A rapid decrease in the pleural fluid fibrinolytic activity to a steady state level occurred with similar k_obs_ (0.40±0.11 and 0.48±0.07 min^−1^) but resulted in different steady‐state levels (2.7 and 1.5%) for PAI‐1‐TFT with sctPA and 2.0 mg/kg DSP in chemically induced and infectious pleural injury, respectively. Further increases in DSP (to 8.0 mg/kg in empyema treatment) did not affect k_obs_ significantly. scuPA based PAI‐1‐TFT with 2.0 mg/kg DSP featured a slower decrease in pleural fluid fibrinolytic activity then that observed with sctPA (0.09±0.06 and 0.16±0.06 min^−1^) and higher steady state fibrinolytic activity (5.6 and 2.5%) for chemically induced and infectious pleural injury, respectively. The observed rate constants of intrapleural accumulation of α‐macroglobulin (αM)/uPA complexes during PAI‐1‐TFT of chemically induced and infectious pleural injury were 0.02±0.01 and 0.03±0.01 min^−1^, respectively (Figure 5i,k), with the maximal intrapleural concentrations of 220±80 and 260±60 nM, respectively. Both plasminogen activating (Figure 5a–d) and fibrinolytic (Figure 5e–h) activities in pleural fluid were suppressed at 24 h after treatment for both successful (GLIS≤10) and unsuccessful (GLIS>10) PAI‐1‐TFT. No DSP was detected by LC MS/MS at 24 h after any intervention (data not shown). Thus, the time needed for effective fibrinolysis (Florova et al., 2015, 2018) in both models of pleural injury is less than 24 h, and likely similar to the one determined previously for TCN‐induced pleural injury in rabbits (6–8 h) (Komissarov et al., 2015). Notably, there was a correlation (*p* < 0.05) between values of fibrinolytic potential (Florova et al., 2015) measured in pleural fluids at the baseline and at 24 h after PAI‐1‐TFT (Figure 5j,l) indicating that fibrinolytic potential reflects status of fibrinolytic system of a specific subject. Intrapleural levels of PAI‐1 (total and active), TGF‐β, TNF‐α, IL‐6, IL‐8 at 24 h after PAI‐1‐TFT in chemically induced and infectious pleural injury are shown in Figure 6a–f. While WBC counts in pleural fluids collected at the time of euthanasia were elevated in both rabbit models, infectious pleural injury was characterized by more than 3‐fold higher total WBCs (data not shown) with a predominance of PMN cells in empyema fluids (Figure 6g). Likewise, this increase was accompanied with an increase in the markers of pleural inflammation comparable to those observed in pleural fluid prior to PAI‐1‐TFT (Figure 1). As shown in Figure 6, statistically significant higher total and active PAI‐1 (Figure 6a,b), TGF‐β, TNF‐α, IL‐6 and IL‐8 (Figure 6c–f) were observed in pleural fluids in *S*. *pneumoniae*‐induced pleural injury. Notably, only treatment with sctPA in combination with 8.0 mg/kg DSP led to a decrease in PMN cells (Figure 6h). However, we did not observe statistically significant changes in the levels of inflammatory markers or PAI‐1 associated with successful PAI‐1‐TFT (Figure 6a–f, green dots). Finally, no apparent local or systemic bleeding was accompanying PAI‐1‐TFT in either model. There was no statistical difference in pleural fluid RBC counts in either model at any dose of DSP alone or in combination with plasminogen activator (Figure 7).

## 4 DISCUSSION

PAI‐1, which is elevated in the pleural space by up to three orders of magnitude in empyema (Aleman et al., 2003; Chung et al., 2005; Philip‐Joet et al., 1995), could be a biomarker of the severity of infectious pleural injury (Chung et al., 2005). Although identified as a major endogenous, mechanism‐based inhibitor of tPA and uPA, to the best of our knowledge, PAI‐1 is not considered a molecular target in fibrinolytic therapy of empyema, possibly because an initial multifold excess of the exogenous plasminogen activator is employed. Nevertheless, the combination of rapid clearance of the drug from the pleural space with constant replenishing of PAI‐1 due to local overexpression dramatically affects the half‐life of intrapleural plasminogen activating activity and fibrinolysis. Low‐dose, local PAI‐1‐TFT allows a significant decrease in the effective therapeutic dose of the drug, minimizing the risk of bleeding complications, and, thereby, expanding the population of patients with empyema that could tolerate pharmacological treatment. The goals of the present study were to validate PAI‐1 as a molecular target in two rabbit models of pleural injury (chemical and infectious) that differ in levels of intrapleural PAI‐1 (Figure 1). To target the PAI‐1 mechanism, we employed DSP, a peptide that interferes with exosite interactions between PAI‐1 and plasminogen activator (Coombs et al., 1998; Madison et al., 1990), thereby competing with PAI‐1 for enzymes (Armstead et al., 2010a, 2010b). Choosing an animal model that adequately recapitulates empyema in humans is critical for translating the results of PAI‐1‐TFT to clinical trials. Unlike mice, rabbis possess a fibrin structure similar to humans, and the biochemistry of rabbit PAI‐1 and other fibrinolytic enzymes and inhibitors is likewise similar (Dewilde et al., 2010; Jo et al., 2009; Klinghofer et al., 2001; Pretorius et al., 2007; Vadivel et al., 2016). Rabbit models allow for testing of human plasminogen activators and have previously been applied successfully in drug development, including the current MIST2 protocol (Rahman et al., 2011) for the treatment of empyema (Dikensoy et al., 2006; Zhu et al., 2006). Here, we demonstrated that DSP mediated PAI‐1‐TFT was effective (i) for both sctPA and scuPA in the treatment of TCN‐induced pleural injury and (ii) for sctPA but not scuPA in infectious pleural injury in rabbits. In both models, the addition of DSP to fibrinolytic therapy rendered low and otherwise ineffective doses of plasminogen activators effective (Figures 2 and 3). Notably, the maximal decrease in the MED of plasminogen activators due to targeting the PAI‐1 mechanism with DSP was 8‐fold in both models, which was similar to the effect of mAbs observed previously in chemically‐induced pleural injury (Florova et al., 2015). The role of PAI‐1 as a molecular target for fibrinolytic therapy in pleural injury is also supported by our previous observations of a decrease in the efficacy of fibrinolytic therapy due to intrapleural stabilization of the active conformation of PAI‐1 (Florova et al., 2018) in a “molecular sandwich” type complex (Florova et al., 2013). Likewise, an increase in the intrapleural level of PAI‐1 in empyema compared to chemically‐induced pleural injury (Figure 1a,b) results in an increase in the severity of the injury, worsening of the outcomes (GLIS=50 of untreated group; Figure 2), and in an increase in the MED of sctPA and scuPA or failure of therapy with doses previously identified as effective in TCN‐induced pleural injury (Komissarov et al., 2016). To achieve efficient PAI‐1‐TFT in empyema, the dose of DSP was also increased from 2.0 mg/kg, which was effective in TCN‐induced pleural injury to 8.0 mg/kg (Figures 2b and 3a). While DSP (8.0 mg/kg) converted ineffective doses of sctPA (0.5 and 0.25 mg/kg) to effective doses (Figures 2 and 3), it did not improve the outcomes of treatment with scuPA in empyema (Figure 2). The striking difference in the effects of DSP on fibrinolytic therapy with scuPA in chemically induced and infectious pleural injury (Figure 2) suggest differences in the mechanisms of uPA‐supported intrapleural fibrinolysis in the two models. The results of LC MS/MS analyses (Figure 4) also demonstrate that an increase in the dose of the adjunct from 2.0 mg/kg results in an increase in the DSP levels in the pleural fluid until PAI‐1‐TFT becomes effective at 8.0 mg/kg DSP (Figure 4, green symbols). Interestingly, DSP alone shows a trend towards improving therapeutic outcomes in both the chemically induced and infectious pleural injury models (Figure 2a), indicating a possible effect on the endogenous fibrinolytic system. Targeting the PAI‐1 mechanism with DSP differs from PAI‐1‐TFT with anti‐PAI‐1 mAbs (Florova et al., 2015, 2018), as DSP interacts with the enzyme rather than PAI‐1 itself. Thus, DSP could be combined with anti‐PAI‐1 mAbs to achieve a further increase in the efficacy of PAI‐1‐TFT due to possible additivity in the effects of different intermolecular mechanisms of modulation of PAI‐1 activity and destabilization of acyl‐enzymes by DSP and mAbs. *S*. *pneumoniae*‐induced empyema in rabbits (Komissarov et al., 2016) recapitulates key features of the disease in humans, including timing and staging: the progression from an early stage, acute empyema to one that is chronic and accompanied by increasing loculation and pleural thickening. An increase in the intrapleural PAI‐1 and cytokines in rabbit empyema, compared to chemically induced pleural injury coincides with an increase in the severity of pleural injury and MED of plasminogen activator (Figures 1 and 6). Likewise, levels of PAI‐1, TNF‐α, TGF‐β, IL‐8 in loculated/complicated pleural effusions in humans are higher than those in transudate/free flowing ones (Aleman et al., 2003; Chung et al., 2005). Although the levels of cytokines differ significantly between chemically induced and infectious pleural injury in rabbits (Figures 1 and 6), PAI‐1 is the molecular target shared by both models, and thus could be a molecular target in human empyema as well. The rate of intrapleural inactivation of uPA and tPA in acute empyema was similar to that which was observed in chemically induced pleural injury (Komissarov et al., 2013) (Figure 5). Although the rates of intrapleural fibrinolysis in two models of pleural injury are comparable, differences in the levels of the molecular target affect the outcomes of PAI‐1‐TFT in both models. In previous studies, we detected high levels of plasminogen activating activity in pleural fluids of patients treated with scuPA at 3 h after fibrinolytic therapy, which was completely suppressed at 23 h (Beckert et al., 2019). Therefore, intrapleural half‐life for plasminogen activators in humans and rabbits are within the same order of magnitude and significantly exceed those reported in the circulation. Thus, an increase in the dose of the plasminogen activator up to 50–100 mg proposed for treatment in advanced empyema (Thommi et al., 2007), could be avoided with low dose PAI‐1‐TFT. Both humans with empyema (Beckert et al., 2019; Florova et al., 2015; Komissarov et al., 2016) and rabbits with infectious pleural injury (Komissarov et al., 2016) (Figure 5), feature inhibition of fibrinolytic activity in pleural fluids collected at baseline and at 23–24 h post intervention. Activation of plasminogen in pleural fluids at baseline results in fibrinolytic activity (Figure 5e–h) that varies up to two orders of magnitude in human samples (Florova et al., 2015; Komissarov et al., 2016). This observation provides support for the concept of fibrinolytic potential (Beckert et al., 2019; Florova et al., 2015; Komissarov et al., 2016), which characterizes the fibrinolytic system of an individual patient that correlates with therapeutic outcomes in humans treated with scuPA (Beckert et al., 2019). Correlation between fibrinolytic potential at the baseline and at 24 h (Figure 5j,l) further supports our hypothesis of a personalized biomarker of an individual's fibrinolytic system. Notably, the level of fibrinolytic activity in pleural fluids collected 10 min or later after treatment (Figure 5e–h) is significantly lower than that observed in the activated baseline pleural fluids collected just prior to injection of plasminogen activator or at 24 h. This could reflect a depletion of pleural fluid plasmin through its binding to intrapleural fibrin, thus increasing the probability of favorable outcomes in individuals with higher fibrinolytic potential (Figures 5j,l and 6). Likewise, a higher steady state (10–40 min) fibrinolytic activity in pleural fluids from animals treated with scuPA based PAI‐1‐TFT (Figure 5e,g) compared to that for sctPA (Figure 5f,h) could indicate less plasmin associated with fibrin and contribute to worse outcomes with scuPA in rabbit empyema (Figure 2). In the injured rabbit (Florova et al., 2015, 2018; Komissarov et al., ,^2009^, ^2013^) and human (Beckert et al., 2019) pleural space, uPA forms “molecular cage” type complexes with endogenous αM, which feature a 10‐fold longer intrapleural half‐life time than free uPA, and could contribute to plasminogen activating activity and fibrinolysis (Florova et al., 2015; Komissarov et al., 2013). Notably, high levels of αM/uPA were detected during PAI‐1‐TFT of both infectious and chemically induced pleural injury and were similar to those observed previously (Komissarov et al., 2013). Surgical interventions are often offered to the majority of patients with advanced stage empyema. However, a significant proportion of patients are poor candidates for surgery due to comorbidities and advanced age. Since conventional intrapleural fibrinolytic therapy confers a risk of bleeding, fluid drainage using negative pressure remains the only option for this group, which has a high mortality rate (Chen et al., 2014; Semenkovich et al., 2018). Our results (Figure 7) clearly indicate that local, low‐dose PAI‐1‐TFT in two models of rabbit pleural injury does not statistically affect the number of RBCs in pleural fluids and, thus, does not increase the risk of intrapleural bleeding complications. Current trends in pharmacological treatment of empyema in humans include (i) de‐escalation of sctPA dose to use in combination with deoxyribonuclease (Hart et al., 2019; Popowicz et al., 2017), (ii) evaluation of need of deoxyribonuclease and dose timing when combined with sctPA (Innabi et al., 2018; Majid et al., 2016, 2017; Mehta et al., 2016; Thommi et al., ,^2012^, ^2014^) and (iii) comparison of the efficacy of tPA and uPA (Aleman et al., 2015; Altmann et al., 2019; Beckert et al., 2019; Bedat et al., 2019; Nie et al., 2014). Our studies demonstrate that the level of PAI‐1 and fibrinolytic potential in pleural fluids (Beckert et al., 2019; Florova et al., 2015; Komissarov et al., 2016) are significant contributors to the outcome of fibrinolytic therapy following pleural injury in two rabbit models. Both parameters could vary up to two orders of magnitude in pleural fluids of human patients with empyema (Aleman et al., 2003; Florova et al., 2015), revealing an opportunity to personalize fibrinolytic therapy. Notably, with use of low dose PAI‐1‐TFT, fibrinolytic potential could become a significant predictor of therapeutic success in patients with empyema. In summary, the similarities between human patients with empyema and our model of empyema in rabbits, raises the possibility of eventual translation of PAI‐1‐TFT to clinical practice. Low‐dose PAI‐1‐TFT was effective and well‐tolerated in two models of pleural injury in rabbits and there was no evidence of intrapleural bleeding, the most serious and commonly encountered complication of intrapleural fibrinolytic therapy of empyema. We envision that the PAI‐1‐TFT therapy presented here could be an effective therapeutic option for a broader population of patients and is safer for those with comorbidities, increased risk of bleeding, or who are otherwise poor candidates for surgical intervention.

## CONFLICTS OF INTEREST

Drs. Komissarov and Florova are supported by NIH and serve as co‐investigators on research involving intellectual property licensed to Lung Therapeutics, Inc. (LTI) and have conflict of interest management plans at The University of Texas Health Science Center at Tyler (UTHSCT). Dr. Idell is likewise supported by NIH and serves as a member of the Board of Directors, Founder and Chief Scientific Officer of LTI. Dr. Idell has an equity position in the company, as does the University of Texas Horizon Fund and UTHSCT. He has a conflict of interest plan acknowledging and managing these declared conflicts of interest through UTHSC. He is an inventor on a patent USPTO # 7332469 held by the UT Board of Regents and licensed to LTI and served on the Safety Review Committee. Dr. Idell also serves as a consultant for Monopar Therapeutics. Drs. Komissarov, Florova and Idell are inventors on patent USPTO # 10,175,255 B2. Messrs. Girard and Sarva serve as research associates on research involving intellectual property licensed to LTI and likewise have conflict of interest management plans at UTHSCT. The rest of the authorship has no conflict of interest to disclose.

## AUTHOR CONTRIBUTIONS

GF, RG, KS, SK, CJDV, DM, MC, KK, and AAK performed experiments and data collection. AOA and AB assisted in animal experiments. AAK, RG, and GF analyzed, plotted, and interpreted the data. AAK, RG, and GF performed statistical analyses. SK edited the manuscript. SI and DBC revised the manuscript. AAK conceived the study, interpreted the data, and wrote the manuscript which was reviewed and approved by all authors.

## Data Availability

Data associated with this study are available from the corresponding author upon reasonable request.

## References

[phy214861-bib-0001] Aleman, C. , Alegre, J. , Monasterio, J. , Segura, R. M. , Armadans, L. , Angles, A. , Varela, E. , Ruiz, E. , & De sevilla, T. (2003). Association between inflammatory mediators and the fibrinolysis system in infectious pleural effusions. Clinical Science, 105, 601–607. 12826021 10.1042/CS20030115

[phy214861-bib-0002] Aleman, C. , Porcel, J. M. , Alegre, J. , Ruiz, E. , Bielsa, S. , Andreu, J. , Deu, M. , Sune, P. , Martinez‐Sogues, M. , Lopez, I. , Pallisa, E. , Schoenenberger, J. A. , Bruno, M. J. , & de Sevilla, T. F. (2015). Intrapleural fibrinolysis with urokinase versus alteplase in complicated parapneumonic pleural effusions and empyemas: A prospective randomized study. Lung, 193, 993–1000. 26423784 10.1007/s00408-015-9807-6

[phy214861-bib-0003] Altmann, E. S. , Crossingham, I. , Wilson, S. , & Davies, H. R. (2019). Intra‐pleural fibrinolytic therapy versus placebo, or a different fibrinolytic agent, in the treatment of adult parapneumonic effusions and empyema. Cochrane Database of Systematic Reviews, 2019(10):CD002312. 10.1002/14651858.CD002312.pub4 PMC6819355 31684683

[phy214861-bib-0004] Armstead, W. M. , Riley, J. , Kiessling, J. W. , Cines, D. B. , & Higazi, A. A. (2010a). Novel plasminogen activator inhibitor‐1‐derived peptide protects against impairment of cerebrovasodilation after photothrombosis through inhibition of JNK MAPK. American Journal of Physiology‐Regulatory, Integrative and Comparative Physiology, 299, R480–R485. 10.1152/ajpregu.00256.2010 PMC2928614 20538898

[phy214861-bib-0005] Armstead, W. M. , Riley, J. , Kiessling, J. W. , Cines, D. B. , & Higazi, A. A. (2010b). PAI‐1‐derived peptide EEIIMD prevents impairment of cerebrovasodilation by augmenting p38 MAPK upregulation after cerebral hypoxia/ischemia. American Journal of Physiology‐Heart and Circulatory Physiology, 299, H76–H80. 20435843 10.1152/ajpheart.00185.2010 PMC2904139

[phy214861-bib-0006] Beckert, L. , Brockway, B. , Simpson, G. , Southcott, A. M. , Lee, Y. C. G. , Rahman, N. , Light, R. W. , Shoemaker, S. , Gillies, J. , Komissarov, A. A. , Florova, G. , Ochran, T. , Bradley, W. , Ndetan, H. , Singh, K. P. , Sarva, K. , & Idell, S. (2019). Phase 1 trial of intrapleural LTI‐01; single chain urokinase in complicated parapneumonic effusions or empyema. JCI Insight, 5, e127470. 10.1172/jci.insight.127470 PMC6542611 30998508

[phy214861-bib-0007] Bedat, B. , Plojoux, J. , Noel, J. , Morel, A. , Worley, J. , Triponez, F. , & Karenovics, W. (2019). Comparison of intrapleural use of urokinase and tissue plasminogen activator/DNAse in pleural infection. ERJ Open Research, 5(3), 00084‐2019. 31528637 10.1183/23120541.00084-2019 PMC6734009

[phy214861-bib-0008] Benedict, C. R. , Refino, C. J. , Keyt, B. A. , Pakala, R. , Paoni, N. F. , Thomas, G. R. , & Bennett, W. F. (1995). New variant of human tissue plasminogen activator (TPA) with enhanced efficacy and lower incidence of bleeding compared with recombinant human TPA. Circulation, 92, 3032–3040. 7586274 10.1161/01.cir.92.10.3032

[phy214861-bib-0009] Carrell, R. W. , Evans, D. L. , & Stein, P. E. (1991). Mobile reactive centre of serpins and the control of thrombosis [published erratum appears in Nature 1993 Aug 19;364(6439):737]. Nature, 353, 576–578. 1922367 10.1038/353576a0

[phy214861-bib-0010] Chaillan‐Huntington, C. E. , Gettins, P. G. W. , Huntington, J. A. , & Patston, P. A. (1997). The P6–P2 region of serpins is critical for proteinase inhibition and complex stability. Biochemistry, 36, 9562–9570. 9236002 10.1021/bi970651g

[phy214861-bib-0011] Chen, K. C. , Chen, H. Y. , Lin, J. W. , Tseng, Y. T. , Kuo, S. W. , Huang, P. M. , Hsu, H. H. , Lee, J. M. , Chen, J. S. , & Lai, H. S. (2014). Acute thoracic empyema: Clinical characteristics and outcome analysis of video‐assisted thoracoscopic surgery. Journal of the Formosan Medical Association, 113, 210–218. 24512757 10.1016/j.jfma.2013.12.010

[phy214861-bib-0012] Chung, C. L. , Chen, C. H. , Sheu, J. R. , Chen, Y. C. , & Chang, S. C. (2005). Proinflammatory cytokines, transforming growth factor‐beta1, and fibrinolytic enzymes in loculated and free‐flowing pleural exudates. Chest, 128, 690–697. 16100155 10.1378/chest.128.2.690

[phy214861-bib-0013] Coombs, G. S. , Bergstrom, R. C. , Madison, E. L. , & Corey, D. R. (1998). Directing sequence‐specific proteolysis to new targets. The influence of loop size and target sequence on selective proteolysis by tissue‐ type plasminogen activator and urokinase‐type plasminogen activator. Journal of Biological Chemistry, 273, 4323–4328. 10.1074/jbc.273.8.4323 9468480

[phy214861-bib-0014] Dewilde, M. , Van De, C. B. , Compernolle, G. , Madsen, J. B. , Strelkov, S. , Gils, A. , & Declerck, P. J. (2010). Subtle structural differences between human and mouse PAI‐1 reveal the basis for biochemical differences. Journal of Structural Biology, 171(1), 95–101. 20230900 10.1016/j.jsb.2010.03.006

[phy214861-bib-0015] Dikensoy, O. , Zhu, Z. , Na, M. J. , Liao, H. , Donnelly, E. , & Light, R. W. (2006). Intrapleural heparin or heparin combined with human recombinant DNase is not effective in the treatment of empyema in a rabbit model. Respirology, 11, 755–760. 17052304 10.1111/j.1440-1843.2006.00934.x

[phy214861-bib-0016] Florova, G. , Azghani, A. , Karandashova, S. , Schaefer, C. , Koenig, K. , Stewart‐Evans, K. , Declerck, P. J. , Idell, S. , & Komissarov, A. A. (2015). Targeting of plasminogen activator inhibitor 1 improves fibrinolytic therapy for tetracycline‐induced pleural injury in rabbits. American Journal of Respiratory Cell and Molecular Biology, 52, 429–437. 25140386 10.1165/rcmb.2014-0168OC PMC4491122

[phy214861-bib-0017] Florova, G. , Azghani, A. O. , Karandashova, S. , Schaefer, C. , Yarovoi, S. V. , Declerck, P. J. , Cines, D. B. , Idell, S. , & Komissarov, A. A. (2018). Targeting plasminogen activator inhibitor‐1 in tetracycline‐induced pleural injury in rabbits. American Journal of Physiology‐Lung Cellular and Molecular Physiology, 314, L54–L68. 28860148 10.1152/ajplung.00579.2016 PMC6048456

[phy214861-bib-0018] Florova, G. , Karandashova, S. , Declerck, P. J. , Idell, S. , & Komissarov, A. A. (2013). Remarkable stabilization of plasminogen activator inhibitor 1 in a “molecular sandwich” complex. Biochemistry, 52, 4697–4709. 23734661 10.1021/bi400470s PMC3855604

[phy214861-bib-0019] Hart, J. A. , Badiei, A. , & Lee, Y. C. G. (2019). Successful management of pleural infection with very low dose intrapleural tissue plasminogen activator/deoxyribonuclease regime. Respirology Case Reports, 7, e00408. 30805192 10.1002/rcr2.408 PMC6373170

[phy214861-bib-0020] Idell, S. , Azghani, A. , Chen, S. , Koenig, K. , Mazar, A. , Kodandapani, L. , Bdeir, K. , Cines, D. , Kulikovskaya, I. , & Allen, T. (2007). Intrapleural low‐molecular‐weight urokinase or tissue plasminogen activator versus single‐chain urokinase in tetracycline‐induced pleural loculation in rabbits. Experimental Lung Research, 33, 419–440. 17994370 10.1080/01902140701703333

[phy214861-bib-0021] Innabi, A. , Surana, A. , Alzghoul, B. , & Meena, N. (2018). Rethinking the doses of tissue plasminogen activator and deoxyribonuclease administrated concurrently for intrapleural therapy for complicated pleural effusion and empyema. Cureus, 10, e2214. 30755840 10.7759/cureus.2214 PMC6368361

[phy214861-bib-0022] Jo, M. , Takimoto, S. , Montel, V. , & Gonias, S. L. (2009). The urokinase receptor promotes cancer metastasis independently of urokinase‐type plasminogen activator in mice. The American Journal of Pathology, 175, 190–200. 19497996 10.2353/ajpath.2009.081053 PMC2708805

[phy214861-bib-0023] Karandashova, S. , Florova, G. , Azghani, A. O. , Komissarov, A. A. , Koenig, K. , Tucker, T. A. , Allen, T. C. , Stewart, K. , Tvinnereim, A. , & Idell, S. (2013). Intrapleural adenoviral delivery of human plasminogen activator inhibitor‐1 exacerbates tetracycline‐induced pleural injury in rabbits. American Journal of Respiratory Cell and Molecular Biology, 48, 44–52. 23002099 10.1165/rcmb.2012-0183OC PMC3547083

[phy214861-bib-0024] Keyt, B. A. , Paoni, N. F. , Refino, C. J. , Berleau, L. , Nguyen, H. , Chow, A. , Lai, J. , Pena, L. , Pater, C. , & Ogez, J. (1994). A faster‐acting and more potent form of tissue plasminogen activator. Proceedings of the National Academy of Sciences of the United States of America, 91, 3670–3674. 8170967 10.1073/pnas.91.9.3670 PMC43643

[phy214861-bib-0025] Klinghofer, V. , Stewart, K. , McGonigal, T. , Smith, R. , Sarthy, A. , Nienaber, V. , Butler, C. , Dorwin, S. , Richardson, P. , Weitzberg, M. , Wendt, M. , Rockway, T. , Zhao, X. , Hulkower, K. I. , & Giranda, V. L. (2001). Species specificity of amidine‐based urokinase inhibitors. Biochemistry, 40, 9125–9131. 11478879 10.1021/bi010186u

[phy214861-bib-0026] Komissarov, A. A. , Florova, G. , Azghani, A. O. , Buchanan, A. , Boren, J. , Allen, T. , Rahman, N. M. , Koenig, K. , Chamiso, M. , Karandashova, S. , Henry, J. , & Idell, S. (2016). Dose dependency of outcomes of intrapleural fibrinolytic therapy in new rabbit empyema models. American Journal of Physiology‐Lung Cellular and Molecular Physiology, 311, L389–L399. 27343192 10.1152/ajplung.00171.2016 PMC5142452

[phy214861-bib-0027] Komissarov, A. A. , Florova, G. , Azghani, A. O. , Buchanan, A. , Bradley, W. M. , Schaefer, C. , Koenig, K. , & Idell, S. (2015). The time course of resolution of adhesions during fibrinolytic therapy in tetracycline‐induced pleural injury in rabbits. American Journal of Physiology‐Lung Cellular and Molecular Physiology, 309, L562–L572. 26163512 10.1152/ajplung.00136.2015 PMC4572422

[phy214861-bib-0028] Komissarov, A. A. , Florova, G. , Azghani, A. , Karandashova, S. , Kurdowska, A. K. , & Idell, S. (2013). Active alpha‐macroglobulin is a reservoir for urokinase after fibrinolytic therapy in rabbits with tetracycline‐induced pleural injury and in human pleural fluids. American Journal of Physiology ‐ Lung Cellular and Molecular Physiology, 305, L682–L692. 23997178 10.1152/ajplung.00102.2013 PMC3840274

[phy214861-bib-0029] Komissarov, A. A. , Mazar, A. P. , Koenig, K. , Kurdowska, A. K. , & Idell, S. (2009). Regulation of intrapleural fibrinolysis by urokinase‐alpha‐macroglobulin complexes in tetracycline‐induced pleural injury in rabbits. American Journal of Physiology‐Lung Cellular and Molecular Physiology, 297, L568–L577. 19666776 10.1152/ajplung.00066.2009 PMC2770793

[phy214861-bib-0030] Madison, E. L. , Goldsmith, E. J. , Gerard, R. D. , Gething, M. J. , Sambrook, J. F. , & Bassel‐Duby, R. S. (1990). Amino acid residues that affect interaction of tissue‐type plasminogen activator with plasminogen activator inhibitor 1. Proceedings of the National Academy of Sciences of the United States of America, 87, 3530–3533. 2110366 10.1073/pnas.87.9.3530 PMC53935

[phy214861-bib-0031] Majid, A. , Kheir, F. , Folch, A. , Fernandez‐Bussy, S. , Chatterji, S. , Maskey, A. , Fashjian, M. , Cheng, G. , Ochoa, S. , Alape, D. , & Folch, E. (2016). Concurrent intrapleural instillation of tissue plasminogen activator and DNase for pleural infection. A single‐center experience. Annals of the American Thoracic Society, 13, 1512–1518. 27333122 10.1513/AnnalsATS.201602-127OC

[phy214861-bib-0032] Majid, A. , Ochoa, S. , Chatterji, S. , Fernandez‐Bussy, S. , Kheir, F. , Rivera, E. , Cheng, G. , & Folch, E. (2017). Safety and efficacy of tissue plasminogen activator and DNase for complicated pleural effusions secondary to abdominal pathology. Annals of the American Thoracic Society, 14, 342–346. 28079399 10.1513/AnnalsATS.201608-594BC

[phy214861-bib-0033] Mehta, H. J. , Biswas, A. , Penley, A. M. , Cope, J. , Barnes, M. , & Jantz, M. A. (2016). Management of intrapleural sepsis with once daily use of tissue plasminogen activator and deoxyribonuclease. Respiration, 91, 101–106. 26761711 10.1159/000443334

[phy214861-bib-0034] Nie, W. , Liu, Y. , Ye, J. , Shi, L. , Shao, F. , Ying, K. , & Zhang, R. (2014). Efficacy of intrapleural instillation of fibrinolytics for treating pleural empyema and parapneumonic effusion: a meta‐analysis of randomized control trials. Clinical Respiratory Journal, 8, 281–291. 10.1111/crj.12068 24428897

[phy214861-bib-0035] Philip‐Joet, F. , Alessi, M. C. , Philip‐Joet, C. , Aillaud, M. , Barriere, J. R. , Arnaud, A. , & Juhan‐Vague, I. (1995). Fibrinolytic and inflammatory processes in pleural effusions. European Respiratory Journal, 8, 1352–1356. 10.1183/09031936.95.08081352 7489803

[phy214861-bib-0036] Popowicz, N. , Bintcliffe, O. , De Fonseka, D. , Blyth, K. G. , Smith, N. A. , Piccolo, F. , Martin, G. , Wong, D. , Edey, A. , Maskell, N. , & Lee, Y. C. G. (2017). Dose de‐escalation of intrapleural tissue plasminogen activator therapy for pleural infection. The alteplase dose assessment for pleural infection therapy project. Annals of the American Thoracic Society, 14, 929–936. 28324671 10.1513/AnnalsATS.201609-673OC

[phy214861-bib-0037] Pretorius, E. , Humphries, P. , Ekpo, O. E. , Smit, E. , & van der Merwe, C. F. (2007). Comparative ultrastructural analyses of mouse, rabbit, and human platelets and fibrin networks. Microscopy Research and Technique, 70, 823–827. 17576129 10.1002/jemt.20482

[phy214861-bib-0038] Rahman, N. M. , Maskell, N. A. , West, A. , Teoh, R. , Arnold, A. , Mackinlay, C. , Peckham, D. , Davies, C. W. , Ali, N. , Kinnear, W. , Bentley, A. , Kahan, B. C. , Wrightson, J. M. , Davies, H. E. , Hooper, C. E. , Lee, Y. C. , Hedley, E. L. , Crosthwaite, N. , Choo, L. , … Davies, R. J. (2011). Intrapleural use of tissue plasminogen activator and DNase in pleural infection. The New England Journal of Medicine, 365, 518–526. 21830966 10.1056/NEJMoa1012740

[phy214861-bib-0039] Semenkovich, T. R. , Olsen, M. A. , Puri, V. , Meyers, B. F. , & Kozower, B. D. (2018). Current state of empyema management. The Annals of Thoracic Surgery, 105, 1589–1596. 29550205 10.1016/j.athoracsur.2018.02.027 PMC5964038

[phy214861-bib-0040] Thommi, G. , Nair, C. K. , Aronow, W. S. , Shehan, C. , Meyers, P. , & McLeay, M. (2007). Efficacy and safety of intrapleural instillation of alteplase in the management of complicated pleural effusion or empyema. American Journal of Therapeutics, 14, 341–345. 17667208 10.1097/01.mjt.0000208275.88120.d1

[phy214861-bib-0041] Thommi, G. , Shehan, C. J. , & McLeay, M. T. (2014). Fibrinolytics in parapneumonic effusions/empyemas. Chest, 146, e103–e104. 25180732 10.1378/chest.14-0706

[phy214861-bib-0042] Thommi, G. , Shehan, J. C. , Robison, K. L. , Christensen, M. , Backemeyer, L. A. , & McLeay, M. T. (2012). A double blind randomized cross over trial comparing rate of decortication and efficacy of intrapleural instillation of alteplase vs placebo in patients with empyemas and complicated parapneumonic effusions. Respiratory Medicine, 106, 716–723. 22398159 10.1016/j.rmed.2012.02.005

[phy214861-bib-0043] Vadivel, K. , Kumar, Y. , Ogueli, G. I. , Ponnuraj, S. M. , Wongkongkathep, P. , Loo, J. A. , Bajaj, M. S. , & Bajaj, S. P. (2016). S2'‐subsite variations between human and mouse enzymes (plasmin, factor XIa, kallikrein) elucidate inhibition differences by tissue factor pathway inhibitor ‐2 domain1‐wild‐type, Leu17Arg‐mutant and aprotinin. Journal of Thrombosis and Haemostasis, 14, 2509–2523. 27797450 10.1111/jth.13538 PMC5504414

[phy214861-bib-0044] van Mourik, J. A. , Lawrence, D. A. , & Loskutoff, D. J. (1984). Purification of an inhibitor of plasminogen activator (antiactivator) synthesized by endothelial cells. Journal of Biological Chemistry, 259, 14914–14921. 6438106

[phy214861-bib-0045] Zhu, Z. , Hawthorne, M. L. , Guo, Y. , Drake, W. , Bilaceroglu, S. , Misra, H. L. , & Light, R. W. (2006). Tissue plasminogen activator combined with human recombinant deoxyribonuclease is effective therapy for empyema in a rabbit model. Chest, 129, 1577–1583. 16778278 10.1378/chest.129.6.1577

